# Mice Deficient in Proglucagon-Derived Peptides Exhibit Glucose Intolerance on a High-Fat Diet but Are Resistant to Obesity

**DOI:** 10.1371/journal.pone.0138322

**Published:** 2015-09-17

**Authors:** Yusuke Takagi, Keita Kinoshita, Nobuaki Ozaki, Yusuke Seino, Yoshiharu Murata, Yoshiharu Oshida, Yoshitaka Hayashi

**Affiliations:** 1 Research Center of Health, Physical Fitness and Sports, Nagoya University, Nagoya, Japan; 2 Department of Genetics, Division of Stress Adaptation and Recognition, Research Institute of Environmental Medicine, Nagoya University, Nagoya, Japan; 3 Department of Metabolic Medicine, Nagoya University School of Medicine, Nagoya, Japan; Hosptial Infantil Universitario Niño Jesús, CIBEROBN, SPAIN

## Abstract

Homozygous glucagon-GFP knock-in mice (*Gcg* ^*gfp/gfp*^) lack proglucagon derived-peptides including glucagon and GLP-1, and are normoglycemic. We have previously shown that *Gcg* ^*gfp/gfp*^ show improved glucose tolerance with enhanced insulin secretion. Here, we studied glucose and energy metabolism in *Gcg* ^*gfp/gfp*^ mice fed a high-fat diet (HFD). Male *Gcg* ^*gfp/gfp*^ and *Gcg* ^*gfp/+*^ mice were fed either a normal chow diet (NCD) or an HFD for 15–20 weeks. Regardless of the genotype, mice on an HFD showed glucose intolerance, and *Gcg* ^*gfp/gfp*^ mice on HFD exhibited impaired insulin secretion whereas *Gcg* ^*gfp/+*^ mice on HFD exhibited increased insulin secretion. A compensatory increase in β-cell mass was observed in *Gcg* ^*gfp/+*^mice on HFD, but not in *Gcg* ^*gfp/gfp*^ mice on the same diet. Weight gain was significantly lower in *Gcg* ^*gfp/gfp*^ mice than in *Gcg* ^*gfp/+*^mice. Oxygen consumption was enhanced in *Gcg* ^*gfp/gfp*^ mice compared to *Gcg* ^*gfp/+*^ mice on an HFD. HFD feeding significantly increased uncoupling protein 1 mRNA expression in brown adipose and inguinal white adipose tissues of *Gcg* ^*gfp/gfp*^ mice, but not of *Gcg* ^*gfp/+*^mice. Treatment with the glucagon-like peptide-1 receptor agonist liraglutide (200 mg/kg) improved glucose tolerance in *Gcg* ^*gfp/gfp*^ mice and insulin content in *Gcg* ^*gfp/gfp*^ and *Gcg* ^*gfp/+*^ mice was similar after liraglutide treatment. Our findings demonstrate that *Gcg* ^*gfp/gfp*^ mice develop diabetes upon HFD-feeding in the absence of proglucagon-derived peptides, although they are resistant to diet-induced obesity.

## Introduction

Diabetes mellitus is characterized by chronic hyperglycemia, and is often associated with obesity. Glucagon, a counter-regulatory hormone to insulin, is one of the most important regulators of glucose homeostasis. It has been recognized that the concerted actions of insulin and glucagon keep plasma glucose levels normal, and that any imbalance in the actions of these molecules can contribute to an increased demand for insulin, impaired glucose tolerance, and persistent hyperglycemia in type 2 diabetes [1]. Under normal physiological conditions, serum glucagon concentration immediately decreases after a meal, whereas in subjects with type 2 diabetes, it can even be found to be elevated after a meal [2–4]. It has therefore been recognized that inappropriate glucagon secretion is at least in part responsible for the pathophysiology of diabetes mellitus [5, 6].

The main physiological role of glucagon is to stimulate hepatic glucose output in order to maintain glucose homeostasis [7]. The physiological role of glucagon in glucose homeostasis has been investigated in animal models where glucagon signaling was blocked at the receptor level by either genetic or pharmacological means: Glucagon receptor knockout mice (*Gcgr* ^-/-^ mice) had lowered plasma glucose levels without exhibiting hypoglycemia [8, 9], whereas treating the animals with glucagon receptor antagonists reduced blood glucose levels in various rodent models [10, 11]. Furthermore, the role of glucagon has been investigated in the pathophysiology of metabolic disorders, where absence of glucagon action was shown to ameliorate diet-induced obesity and diabetes. For example, pharmacological blockage of glucagon receptor signaling was shown to improve glucose metabolism in animals that were fed a high-fat diet (HFD) [12]. Similarly, *Gcgr* ^-/-^ mice were found to be resistant to HFD-induced obesity because of reduced energy intake [13]. However, both glucagon and glucagon-like peptide-1 (GLP-1) are derived from a common precursor, proglucagon, and these animals exhibited elevated levels of circulating glucagon-like peptide-1 (GLP-1) as a consequence to compensate glucagon deficiency. Therefore, the role of glucagon and GLP-1 and their contribution to the pathophysiology of diabetes and obesity remain to be fully clarified.

Obesity is now recognized as a major epidemic and is associated with diseases such as metabolic syndrome, type 2 diabetes mellitus, and atherosclerotic cardiovascular disease [14]. Disturbing the balance between energy intake and energy expenditure plays a crucial role in the pathogenesis of obesity. The capacity for increased thermogenesis through brown adipose tissue (BAT) activation is important for body weight homeostasis [15]. In addition, the development of inducible brown-like adipocytes (also referred to as “brite” or “beige” adipocytes), in white adipose tissue (WAT) depots in response to certain stimuli has recently been reported, suggesting that these adipocytes have the potential to tilt the energy balance from storage to expenditure [16]. In response to various physiological and pharmacological stimuli, the expression of brown-fat-like genes such as uncoupling protein 1 (*Ucp1*), cell death-inducing DFFA-like effector a (*Cidea*) and deiodinase iodothyronine type II (*Dio2*) are induced in WAT. These changes in expression patterns contribute to adaptive thermogenesis and energy expenditure similar to those seen in classical BAT [16]. Glucagon is also involved in the control of energy expenditure and thermogenesis [17, 18].

We recently reported that homozygous glucagon-GFP knock-in mice (*Gcg* ^*gfp/gfp*^), which lack not only glucagon but also GLP-1 and GLP-2, are normoglycemic and display improved glucose tolerance with enhanced insulin secretion upon acute glucose loading [19, 20]. *Gcg* ^*gfp/gfp*^ mice exhibited cold intolerance and impaired thermogenesis in response to a cold stimulus [21]. To further understand the roles of proglucagon-derived peptides (PGDPs) in the pathophysiology of obesity and diabetes, we examined glucose and energy metabolism in *Gcg* ^*gfp/gfp*^ mice fed HFD.

## Methods

### Animal studies

This study was performed according to a protocol approved by the Nagoya University Institutional Animal Care and Use Committee. The establishment of the glucagon-GFP knock-in mice backcrossed with C57BL/6J background for at least 12 generations have been previously described [20]. The mice were housed in a temperature-controlled room under a standard 12-h light/dark cycle. Five-week-old *Gcg* ^*gfp/gfp*^ and *Gcg* ^*gfp/+*^male mice were divided into two groups and fed either a normal chow diet (NCD, 12.6% energy content of fat; CE-2 from CLEA Japan, Osaka, Japan) or a high-fat diet (HFD, 56.7% energy content of fat; HFD32 from CLEA Japan) for 15–20 weeks. For GLP-1 supplementation experiments, GLP-1 receptor agonist liraglutide (200 mg/kg) was subcutaneously administered once daily during the final 4 weeks of HFD-feeding. Tissue samples were collected from mice that were fasted for 16 h or from mice that were fed for 6 h after a 16-h starvation period. *Gcg* ^*gfp/+*^ mice were used as control in the present study, as *Gcg* ^*gfp/+*^ and *Gcg* ^*+/+*^ mice do not differ in terms of glucose tolerance and hepatic gene expression patterns [19, 20, 22, 23].

### Glucose tolerance test and insulin tolerance test

These tests were performed on mice that were fed a NCD or an HFD for 15 weeks, as described previously [24]. In brief, mice were deprived of food for 16 h, and 2 g/kg body weight of glucose was intraperitoneally administered (IPGTT). IPGTT in liraglutide-treated mice was performed after treatment for 2 weeks. For insulin tolerance test (ITT), five hours after food depletion and immediately before the experiments were carried out insulin was injected at a dose of 0.75 U/kg. Blood was collected at multiple time intervals to measure glucose and insulin levels by subzygomatic approach using goldenrod animal lancet. Alternatively, tale tip bleeding was also employed when only glucose level was measured.

### Islet isolation, measurement of insulin, and morphometric analysis

Pancreatic islets were isolated using the collagenase digestion method and glucose-induced insulin secretion from isolated islets were carried out as described previously [19]. Briefly, sets of 5 islets similar in size were hand-picked from pooled isolated islets and incubated in the Krebs-Ringer buffer containing 2.8 mmol/L glucose for 30 min, groups of five islets were incubated in the Krebs-Ringer buffer containing 16.7 mmol/L glucose for 30 min. Insulin concentration in the buffer was analyzed and normalized to cellular insulin content. Immunohistochemistry and morphometric analysis were performed as described previously [19]. Briefly, the pancreata were fixed in 4% paraformaldehyde and embedded in paraffin. Tissue sections were incubated overnight at 4°C with primary antibodies against insulin (1:1000; Abcam plc, Cambridge, UK) or Ki-67 (1:250; Abcam) followed by a 90-min incubation with Alexa Fluor-conjugated secondary antibody (1:500; Alexa Fluor 488 or 1:1000, Alexa Fluor 568, Invitrogen, Grand Island, NY, USA) at room temperature. Images were taken using an HS BZ-9000 fluorescence microscope system (Keyence Corp., Osaka, Japan). For morphometric analyses, serial sections of 4–5 μm thickness were cut from each paraffin block at 200-μm intervals, and 4–5 sections were randomly selected from each mouse and immunostained for insulin and Ki-67. The insulin-positive cell areas relative to the sectional area or the number of Ki-67-positive cells relative to insulin-positive islet cells (3890±540 cells per mouse) were determined by the HS BZ-II analysis application and Image J software.

### Determination of BAT triglyceride content and histological analysis in BAT

BAT triglyceride content was measured as previously described [24]. The BAT was fixed in 4% paraformaldehyde, and then embedded in paraffin. 4 μm sections were stained with hematoxylin andeosin (H&E staining). Representative images of H&E stained sections were captured using an Olympus BX53 system (Olympus Corporation, Tokyo, Japan).

### Biochemical analysis

Blood glucose levels were measured with Antisense III (Horiba Ltd., Kyoto, Japan). Plasma insulin levels were determined using a mouse insulin enzyme-linked immunosorbent assay kit (Morinaga-Seikagaku Co. Ltd., Yokohama, Japan). Insulin in the medium and its content was determined using the HTRF insulin assay kit (Cisbio Bioassays, Bagnols-sur-Cèze, France).

### Energy balance

A comprehensive animal metabolic monitoring system (CLAMS; Columbus Instruments, Columbus, OH, USA) was used for 3 days. Energy expenditure and respiratory exchange ratios (RER) were calculated by measuring gas exchange rates. The respiratory exchange ratio was computed as carbon dioxide output (*VCO*_*2*_) divided by oxygen consumption (*VO*_*2*_). Physical activity was measured on the *x* axes by counting the number of brakes in an infrared beam during a measurement period.

### Isolation of tissue RNA and quantitative real-time RT-PCR

Total RNA was extracted from isolated islets, BAT and iWAT using RNAiso Plus reagent (Takara Bio Inc., Shiga, Japan) or the RNeasy Plus Kit (Qiagen Inc., Valencia, CA, USA). Complementary DNA was synthesized using a PrimeScript RT Master Mix (Takara Bio Inc.). Quantitative real-time RT-PCR was performed with the StepOne real-time PCR Systems (Applied Biosystems, Foster City, CA, USA) by using a THUNDERBIRD SYBR qPCR Mix (TOYOBO Co. Ltd, Osaka, Japan). The sequences of the primers used for the analyses are available upon request.

### Statistical analysis

Data are presented as means ± SEM. Significance was evaluated using Student’s *t* test or ANOVA followed by post-test comparisons when applicable. Energy expenditure was analyzed by using ANCOVA with body weight as the covariate. A *p* value < 0.05 was regarded as statistically significant.

## Results

### Glucose metabolism

**Fig 1:**
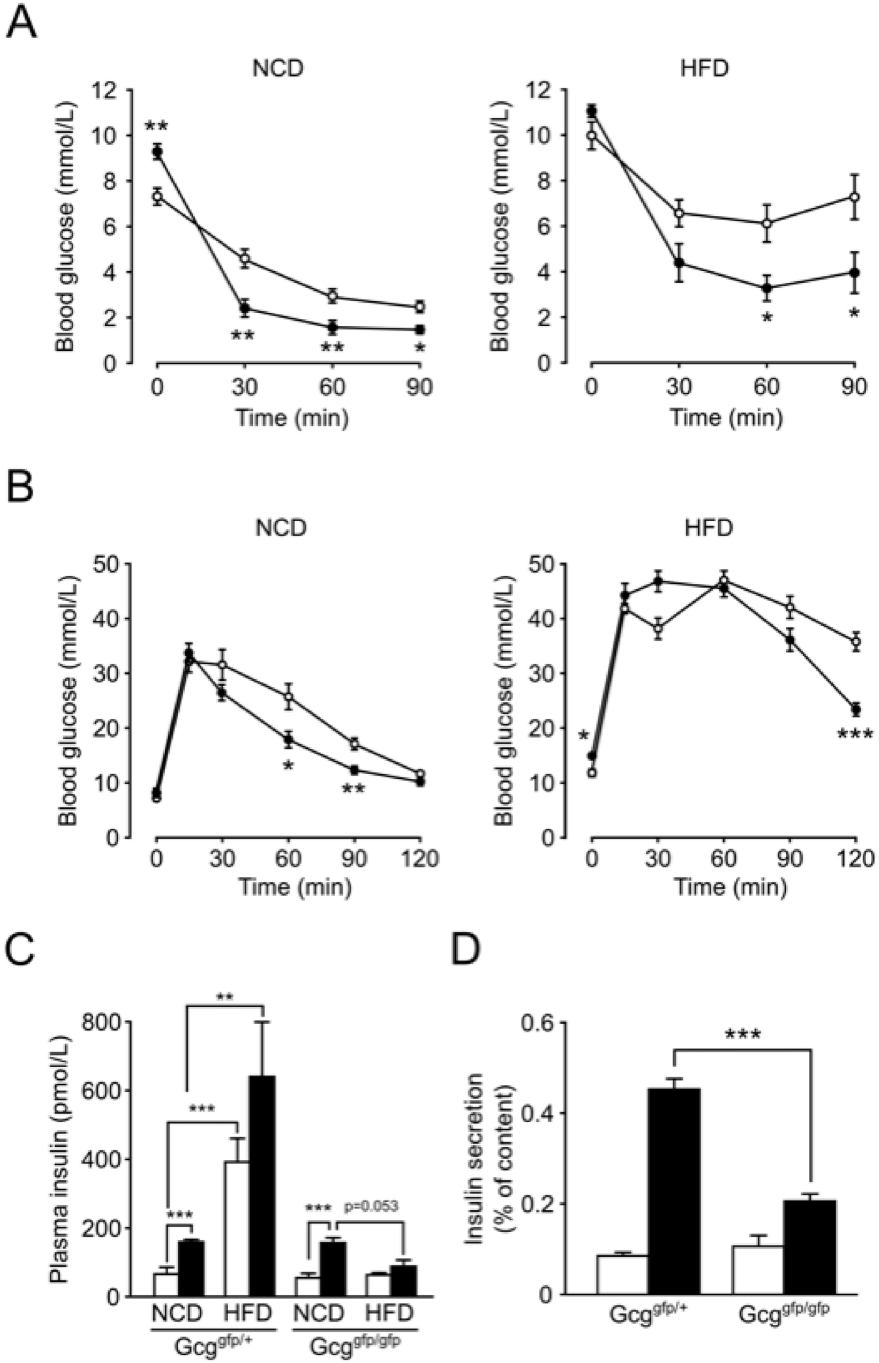
*Gcg* ^*gfp/gfp*^ mice fed HFD exhibit glucose intolerance and impaired insulin secretion. (A) Insulin tolerance test. Open circles, *Gcg* ^*gfp/+*^ mice; closed circles, *Gcg* ^*gfp/gfp*^ mice (n = 5–7). *p < 0.05; **p < 0.01. (B) Blood glucose levels during IPGTT. Open circles, *Gcg* ^*gfp/+*^ mice; closed circles, *Gcg* ^*gfp/gfp*^ mice (n = 4–6). *p < 0.05; **p < 0.01; ***p < 0.001. (C) Plasma insulin levels at 0 min (white bars) and 15 min (black bars) after i.p. glucose loading (n = 4–6). **p < 0.01; ***p < 0.001. (D) Glucose-induced insulin secretion from isolated islets. Isolated islets were stimulated by 2.8 mmol/L glucose (white bars) or 16.7 mmol/L glucose (black bars) for 30 min. Insulin secretion is expressed as the ratio of insulin released into the medium relative to insulin content (n = 6–12). ***p < 0.001. Data are presented as means ± SEM.

*Gcg* ^*gfp/gfp*^ and *Gcg* ^*gfp/+*^ mice were fed a NCD or an HFD for 15 weeks beginning at 5 weeks after birth. While decreases in blood glucose levels in response to insulin administration was impaired in both mice on an HFD (NCD vs HFD in both *Gcg* ^*gfp/+*^ and *Gcg* ^*gfp/gfp*^: p<0.05 at 0, 30, 60 and 90 min after insulin administration), *Gcg* ^*gfp/gfp*^ mice exhibited lower glucose levels compared to *Gcg* ^*gfp/+*^ mice, indicating that insulin resistance in *Gcg* ^*gfp/gfp*^ mice was much less severe than in *Gcg* ^*gfp/+*^ mice (Fig 1A). Glucose tolerance, evaluated by IPGTT, was markedly impaired in both *Gcg* ^*gfp/+*^ and *Gcg* ^*gfp/gfp*^ mice on HFD (Fig 1B). In mice fed NCD, blood glucose levels in *Gcg* ^*gfp/gfp*^ was significantly lower compared to *Gcg* ^*gfp/+*^ at 30 and 60 minutes after glucose load. However, in mice fed HFD, no significant difference in blood glucose levels were observed between *Gcg* ^*gfp/+*^ and *Gcg* ^*gfp/gfp*^ at the same time points. Plasma insulin levels measured at 0 and 15 min after glucose loading were significantly increased in *Gcg* ^*gfp/+*^ mice on HFD compared to those on NCD, whereas such increase was not observed in *Gcg* ^*gfp/gfp*^ mice (Fig 1C). We further examined glucose-induced insulin secretion from isolated islets of HFD-fed mice, finding it to be significantly reduced in *Gcg* ^*gfp/gfp*^ mice in comparison with *Gcg* ^*gfp/+*^ mice (Fig 1D). These results suggest that HFD-induced changes in β-cell function are attenuated in *Gcg* ^*gfp/gfp*^ mice deficient in PGDPs.

**Fig 2:**
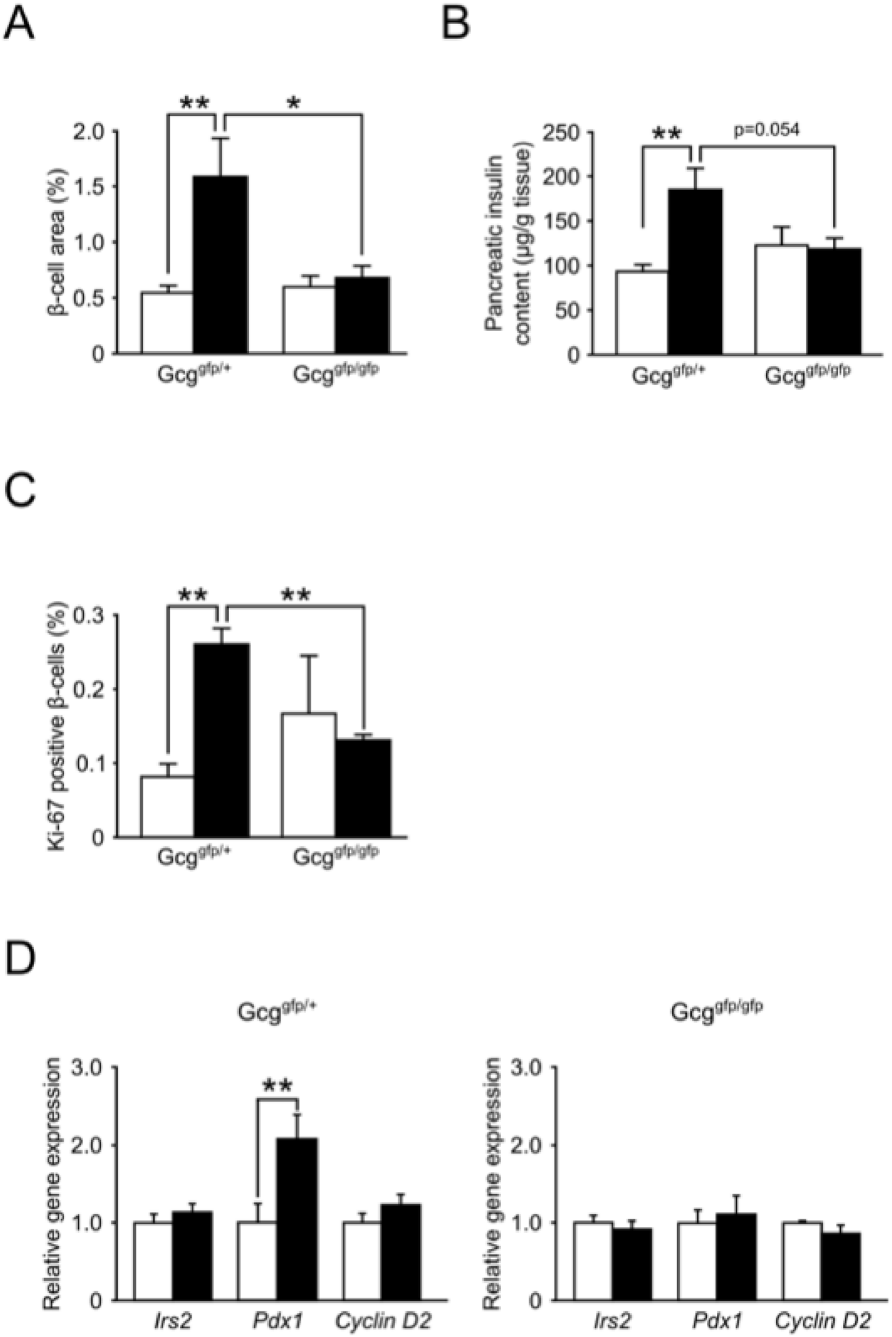
*Gcg* ^*gfp/gfp*^ mice fed HFD fail to show β-cell expansion and increased insulin contents. (A) β-cell area is shown as insulin-positive area relative to total pancreas area by morphometric analysis (n = 5–6). (B) Insulin contents in pancreata (n = 6–9). (C) Proliferation of pancreatic β-cells. Percentage of Ki-67-positive β-cells is shown as the number of Ki-67-positive cells relative to insulin-positive islet cells. White bars, mice fed NCD; black bars, mice fed HFD (n = 3–4). **p < 0.01. (D) mRNA Expression of *Irs2*, *Pdx1*, and *cyclin D2* in islets. White bars, mice fed NCD; black bars, mice fed HFD (n = 4–8). *p < 0.05; **p < 0.01. Data are presented as means ± SEM.

To clarify the cause for impaired insulin secretion in *Gcg* ^*gfp/gfp*^ mice, we analyzed the morphology of pancreatic islets and their gene expression. Morphometric analysis revealed that HFD-feeding increased β-cell area in *Gcg* ^*gfp/+*^ mice, but failed to increase β-cell area in *Gcg* ^*gfp/gfp*^ mice (Fig 2A and S1 Fig). Pancreas weight and β-cell mass are shown in S2 and S3 Figs, and these data showed that no significant increase in β-cell mass was induced by HFD-feeding in *Gcg* ^*gfp/gfp*^ mice. In addition, HFD-feeding resulted in a 2-fold increase of insulin content in pancreata of *Gcg* ^*gfp/+*^ mice, whereas this increase was diminished in *Gcg* ^*gfp/gfp*^ mice (Fig 2B). We performed immunostaining for Ki-67 to estimate β-cell proliferation, and observed an increase in Ki-67-positive β-cells in *Gcg* ^*gfp/+*^ mice on an HFD, but not in *Gcg* ^*gfp/gfp*^ mice on the same diet (Fig 2C). Expression of the pancreatic duodenal homeobox-1 (*Pdx1*) gene, which is an important transcription factor for β-cell proliferation and survival, was up-regulated in islets of *Gcg* ^*gfp/+*^ mice on an HFD. By contrast, up-regulation of *Pdx1* expression by HFD-feeding was not observed in islets of *Gcg* ^*gfp/gfp*^ mice (Fig 2D). Expression of cyclin D2 and insulin receptor substrate-2 (*Irs2*) did not change significantly by HFD-feeding (Fig 2D).

### Body weight and energy metabolism

**Fig 3:**
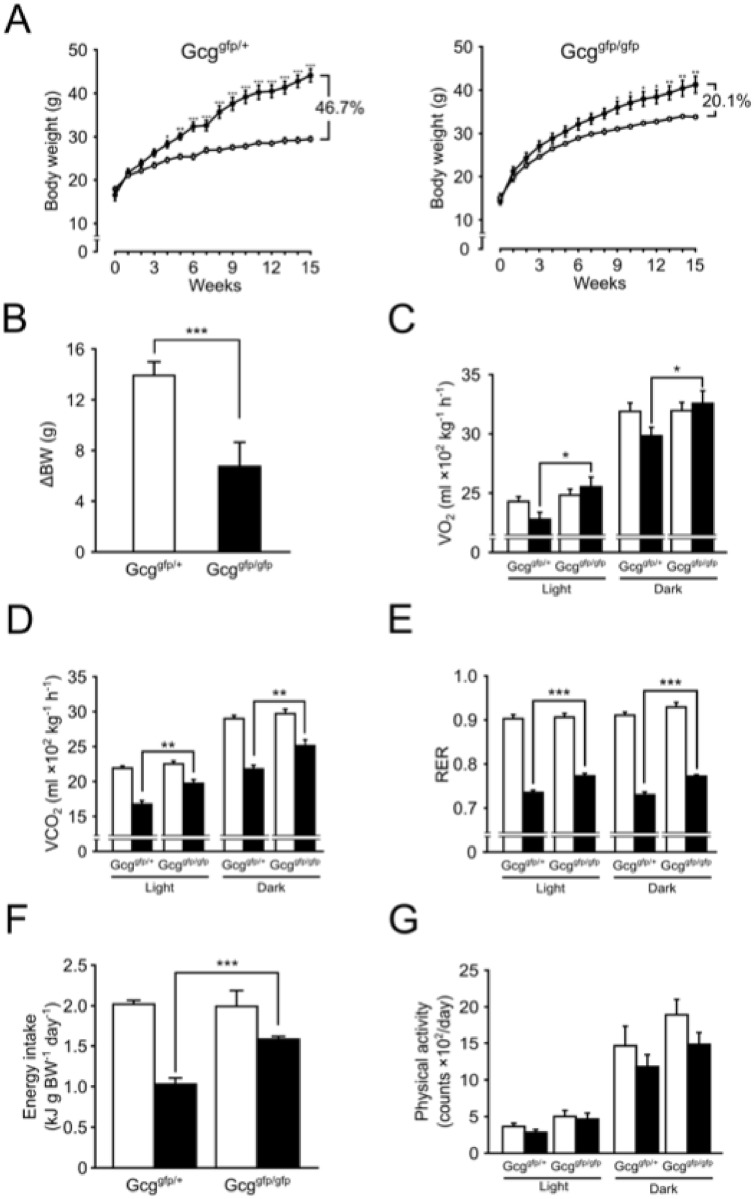
Changes in body weight and quantification of energy expenditure. Indirect calorimetry was analyzed by using CLAMS. (A) Body weight changes during HFD feeding. (B) Body weight gain at 15 weeks of HFD-feeding. (C) Oxygen consumption (*VO*_*2*_). (D) Carbon dioxide output (*VCO*_*2*_). (E) Respiratory exchange rates (RER). (F) Energy intake. (G) Physical activity. White bars, mice fed NCD; black bars, mice fed HFD (n = 6–8). *p < 0.05; **p < 0.01; ***p < 0.001. Data are presented as means ± SEM.

Although both *Gcg* ^*gfp/gfp*^ and *Gcg* ^*gfp/+*^ mice on HFD showed an increase in body weight throughout the experimental period, the ratio of weight gain in *Gcg* ^*gfp/gfp*^ mice was lower than in *Gcg* ^*gfp/gfp*^ mice (46.7% vs. 20.1%, Fig 3A). S4 Fig depicts difference in body weight between mice fed NCD and those fed HFD. Body weight gain of *Gcg* ^*gfp/+*^ mice at 15 weeks of HFD-feeding was greater than that of *Gcg* ^*gfp/gfp*^ mice (Fig 3B). In concordance with our previous study [23], under NCD-feeding, both mice showed comparable levels of energy intake, physical activity, *VO*_*2*_, *VCO*_*2*_ and RER (Fig 3C-3E). However, *Gcg* ^*gfp/gfp*^ mice on HFD exhibited higher levels of *VO*_*2*_, *VCO*_*2*_ and RER compared to *Gcg* ^*gfp/+*^ mice on the same diet (Fig 3C-3E). Although energy intake was suppressed in HFD-fed mice relative to the NCD-fed mice, under HFD-feeding, energy intake in *Gcg* ^*gfp/gfp*^ mice was significantly higher than in *Gcg* ^*gfp/+*^ mice (Fig 3F). These results in addition to the observation that HFD-feeding did not alter physical activity in either mice (Fig 3G), indicate that *Gcg* ^*gfp/gfp*^ mice retained normal energy expenditure even under HFD-feeding.

### Analysis of adipose tissue

**Fig 4:**
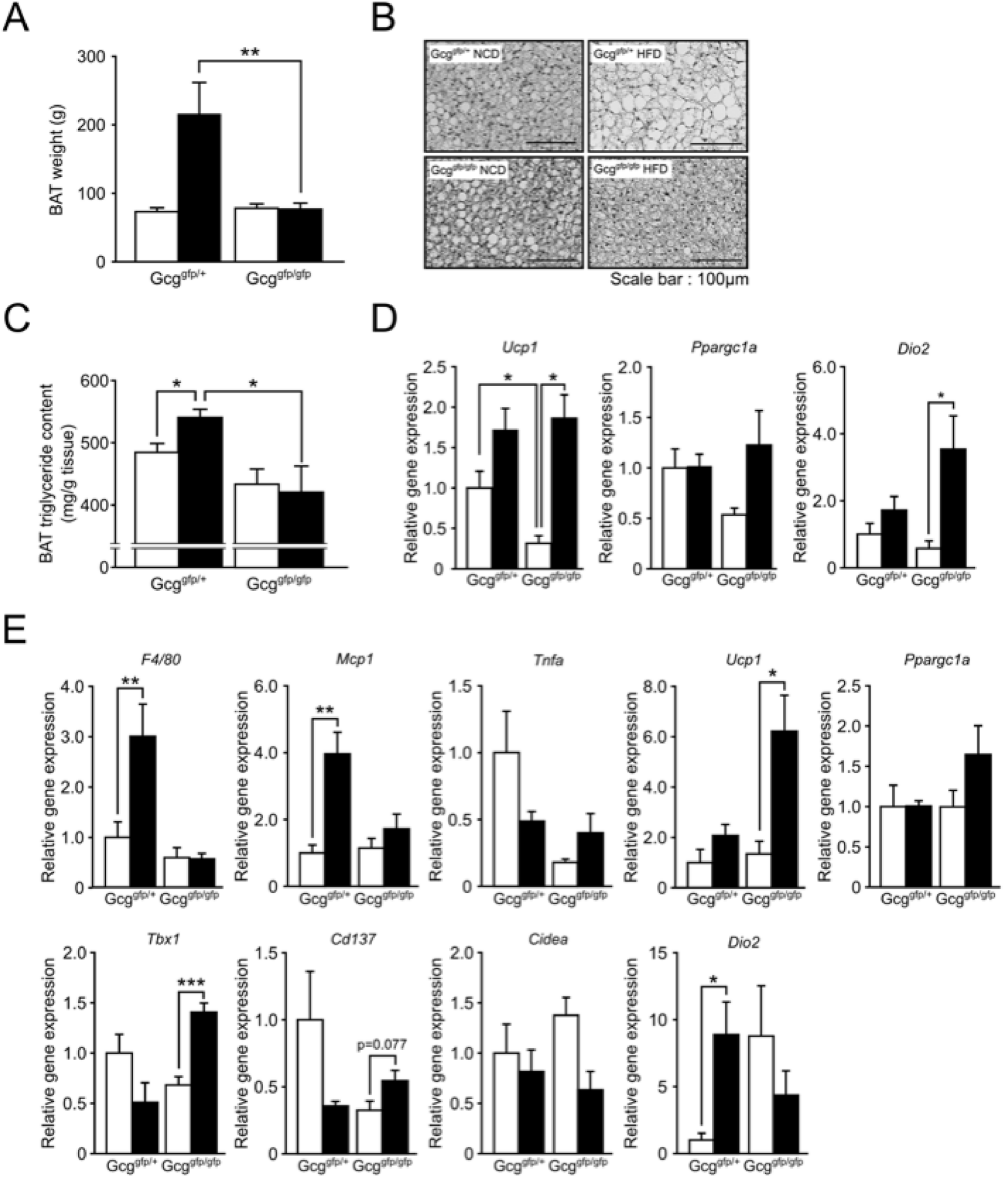
Analysis of adipose tissue with regard to energy metabolism. (A) Intrascapular BAT weights after NCD- or HFD-feeding (n = 6–8). (B) Representative H&E staining of BAT. (C) Triglyceride contents in BAT (n = 4). (D) mRNA expression of *Ucp1*, *Ppargc1a*, and *Dio2* in BAT (n = 5–8). (E) mRNA expression of *Ucp1*, *Ppargc1a*, *Dio2*, *F4/80*, *Mcp1*, *Tnfa*, *Tbx1*, *Cd137*, and *Cidea* in inguinal WAT. White bars, mice fed on a NCD; black bars, mice fed on an HF diet (n = 4–5). *p < 0.05; ***p < 0.001. Data are presented as means ± SEM.

Given the crucial role of BAT in energy expenditure, we next analyzed histology and gene expression in intrascapular BAT. BAT weight in *Gcg* ^*gfp/gfp*^ mice did not increase by HFD-feeding, whereas HFD-feeding increased BAT weight in *Gcg* ^*gfp/+*^ mice (Fig 4A). Histological analysis revealed smaller fat droplets and significantly lower triglyceride content in BAT of *Gcg* ^*gfp/gfp*^ mice on HFD than in that of *Gcg* ^*gfp/+*^ mice on the same diet (Fig 4B and 4C). Under NCD-feeding, *Ucp1* mRNA expression in *Gcg* ^*gfp/gfp*^ BAT was significantly lower than that in *Gcg* ^*gfp/+*^ BAT, while *Dio2* mRNA expression was comparable between the both mice (Fig 4D). By contrast, HFD-feeding induced a significant increase in *Ucp1* and *Dio2* mRNA only in BAT of *Gcg* ^*gfp/gfp*^ mice (Fig 4D). These results indicate that accumulation of lipid in BAT induced by HFD-feeding was attenuated in *Gcg* ^*gfp/gfp*^ mice and that expenditure of lipids as an energy source was increased in the BAT of *Gcg* ^*gfp/gfp*^ mice on HFD.

We also examined gene expression in inguinal white adipose tissue (iWAT) because brown-like adipocytes have the potential to tilt the energy balance from storage to expenditure [16]. We found that HFD-feeding induced mRNA expression of *Ucp1*, and *Ppargc1a*, but not *Cidea* (the classical BAT marker), in iWAT (Fig 4E). In addition, HFD-feeding induced the expression of *Tbx1* and *Cd137* (molecular markers of brown-like adipocytes) in *Gcg* ^*gfp/gfp*^ mice but not in *Gcg* ^*gfp/+*^ mice. These results indicate that HFD-feeding differentially alters gene expression in iWAT. On the other hand, expression of *F4/80* and *Mcp-1* was induced by HFD feeding in *Gcg* ^*gfp/+*^ mice but not in *Gcg* ^*gfp/gfp*^ mice, suggesting macrophages infiltration in *Gcg* ^*gfp/+*^. These results suggest that, similar to BAT, energy expenditure in iWAT is also enhanced in *Gcg* ^*gfp/gfp*^ mice.

### Treatment with GLP-1 receptor agonist liraglutide

**Fig 5:**
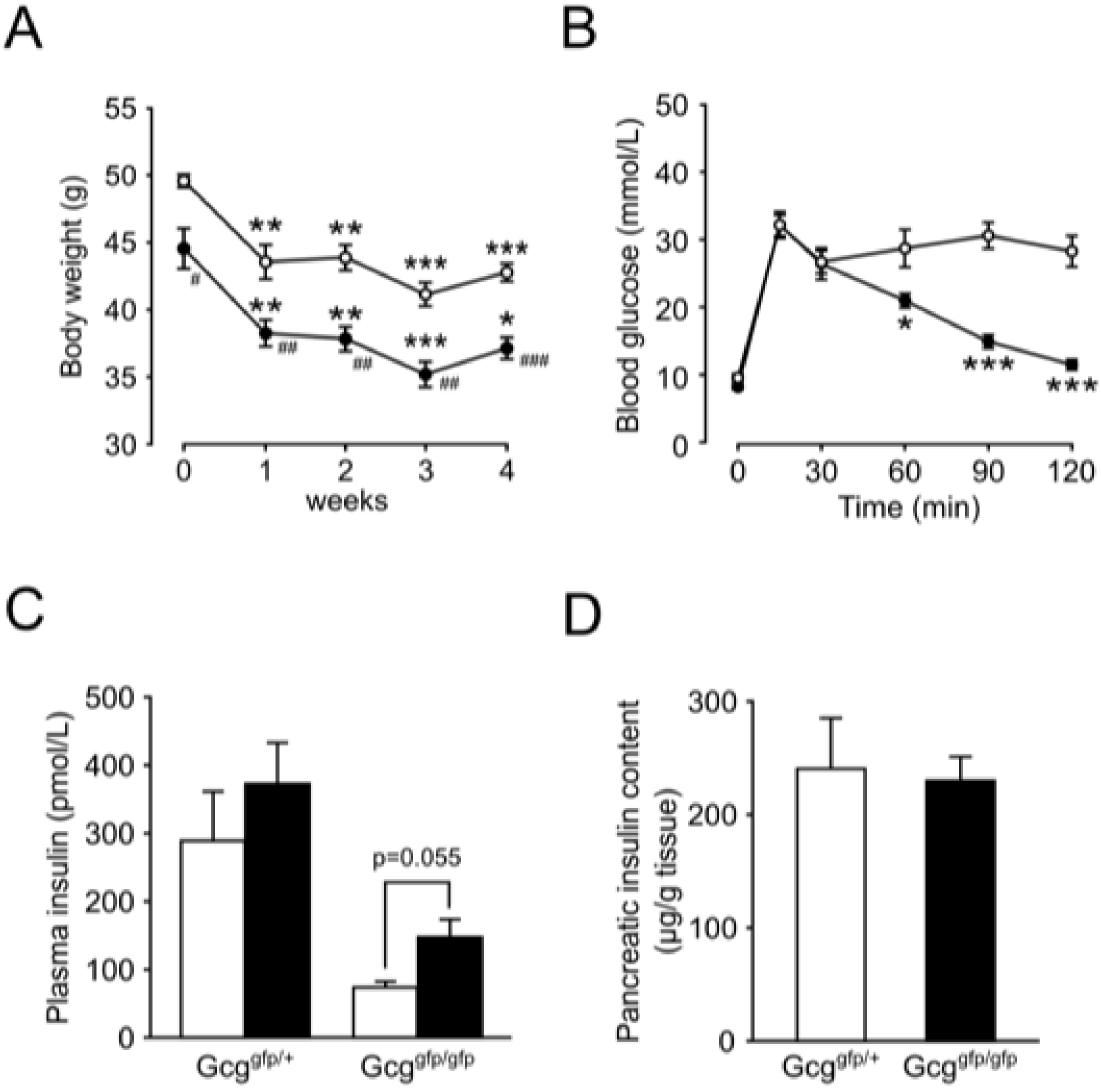
Effect of liraglutide on glucose metabolism. Liraglutide (200 mg/kg) was subcutaneously administered once a day for last 4 weeks of HFD-feeding. (A) body weights during liraglutide treatment (n = 6–7). Open circles, liraglutide-treated *Gcg* ^*gfp/+*^ mice on HFD; closed circles, liraglutide-treated *Gcg* ^*gfp/gfp*^ mice on HFD. **p < 0.01 vs *Gcg* ^*gfp/+*^ mice. ^#^p < 0.05; ^##^p < 0.01 vs. before liraglutide treatment. (B) Blood glucose levels during IPGTT (n = 6–7). Open circles, liraglutide-treated *Gcg* ^*gfp/+*^ mice on HFD; closed circles, liraglutide-treated *Gcg* ^*gfp/gfp*^ mice on HFD. *p < 0.05; ***p < 0.001 vs. *Gcg* ^*gfp/+*^ mice. (C) Plasma insulin levels 0 min (white bars) and 15 min (black bars) after i.p. glucose loading (n = 6–7). (D) Insulin contents in pancreata. White bars, liraglutide-treated *Gcg* ^*gfp/+*^ mice on HFD; black bars, liraglutide-treated *Gcg* ^*gfp/gfp*^ mice on HFD (n = 5–7). Data are presented as means ± SEM.

GLP-1 is known to stimulate not only insulin secretion but also β-cell proliferation both *in vivo* and *in vitro* [25]. To clarify whether the absence of GLP-1 is responsible for impaired β-cell function in *Gcg* ^*gfp/gfp*^ mice, effect of GLP-1 receptor agonist liraglutide administration at 200 μg/kg, once daily during the last 4 weeks of HFD-feeding, was analyzed. Treatment with liraglutide significantly reduced body weights in both *Gcg* ^*gfp/+*^ and *Gcg* ^*gfp/gfp*^ mice on an HFD (Fig 5A). The peak blood glucose levels during IPGTT were 32.1±1.6 and 32.1±1.9 in liraglutide-treated *Gcg* ^*gfp/+*^ and *Gcg* ^*gfp/gfp*^ mice, respectively (Fig 5B). As those in HFD-fed *Gcg* ^*gfp/+*^ and *Gcg* ^*gfp/gfp*^ mice were 47.1±1.7 and 46.8±1.9 respectively (see Fig 1B), liraglutide significantly improved glucose tolerance in these mice (p<0.001 and <0.001 in *Gcg* ^*gfp/+*^ and *Gcg* ^*gfp/gfp*^, respectively). Furthermore, blood glucose levels in liraglutide-treated *Gcg* ^*gfp/+*^ mice were significantly lower than liraglutide-treated *Gcg* ^*gfp/gfp*^ mice at time points later than 60 min after glucose administration (Fig 5B). These results suggest that *Gcg* ^*gfp/gfp*^ mice are more responsive to GLP-1 administration than the control mice. Although no significant increase in plasma insulin levels to glucose load was observed in liraglutide-treated, HFD-fed *Gcg* ^*gfp/gfp*^ (Fig 5C), insulin content in these animals was 230.0±21.1 μg/g tissue, which was significantly greater (p< 0.01) than 118.3 ±12.3 μg/g tissue in HFD-fed *Gcg* ^*gfp/gfp*^ mice (Figs 2B and 5D). Collectively, GLP-1 agonist markedly ameliorated metabolic changes caused by HFD-feeding in the *Gcg* ^*gfp/gfp*^ mice.

## Discussion

Compared to insulin, glucagon has long been dismissed as a minor contributor to diabetes. However, it was recently reported that destruction of β-cells by treatment with streptozotocin in *Gcgr* ^*-/-*^ mice failed to increase blood glucose levels [26]. Glucagonocentrism was proposed as a new framework to facilitate such results whereby a lack of insulin was proposed to lead to glucagon excess, in turn causing diabetes abnormalities [27]. In the present study, we showed that in spite of a glucagon deficiency, HFD-fed *Gcg* ^*gfp/gfp*^ mice did develop diabetes, which was due to impaired insulin secretion and not insulin resistance. Our results thus indicate that glucagon is not essential for pathogenesis of HFD-induced diabetes and that secondary increases in GLP-1 should play roles in resistance to develop diabetes in other glucagon-deficient models.

Under an insulin-resistant or hyperglycemic condition, β-cell compensates for an increased insulin demand by increasing its capacity for secretion and by expanding its mass [28, 29]. In the present study, we found that such compensation is defective in *Gcg* ^*gfp/gfp*^ mice fed HFD; no increase in capacity of insulin secretion nor any β-cell expansion was observed in these mice. In contrast to *Gcg* ^*gfp/gfp*^ mice, *Gcgr* ^*-/-*^ mice showed improved glucose tolerance upon oral GTT and IPGTT treatment, when compared with wild type mice on HFD [13]. It has also been shown that administration of glucagon receptor antagonist for 24–30 days to mice on HFD enhances insulin secretion, increases β-cell mass, and improves glucose tolerance [12]. Except for *Gcg* ^*gfp/gfp*^ mice, these glucagon-deficient models exhibits increased GLP-1 levels in circulation [13, 30]. GLP-1 is known to stimulate not only insulin secretion but also β-cell expansion by acting as a growth factor both in experimental animal models as well as cultured β-cells, promoting their proliferation, survival, and differentiation [25]. Therefore absence of GLP-1 in *Gcg* ^*gfp/gfp*^ mice should account for defective β-cell compensation.

Indeed, *Glp1r* ^-/-^ mice fed HFD exhibited no significant increase in pancreatic insulin content and β-cell mass [31]. Jun et al recently demonstrated that loss of GLP-1 action aggravates glucose tolerance in mice deficient in glucagon receptor [32]. In addition, restoration of islet GLP-1 receptor expression enhanced exendin-4-induced β-cell proliferation and expansion of β-cell mass in *Glp1r* ^*-/-*^ mice on HFD [33]. Thus, the findings in models with antagonized glucagon action can be explained by elevated GLP-1 levels in circulation. Omar et al. recently reported that treatment with GLP-1 receptor antagonist Exendin 9–39 in streptozotocin-treated *Gcgr* ^*-/-*^ mice significantly increased the glycemic excursion during OGTT [34]. It is in this context that we assumed that administration of GLP-1 receptor agonist liraglutide should improve β-cell function in *Gcg* ^*gfp/gfp*^ mice on an HFD. Indeed, liraglutide treatment for 4 weeks enhanced insulin secretion and pancreatic insulin content in HFD-fed *Gcg* ^*gfp/gfp*^ mice. These results therefore indicate that GLP-1 deficiency contributes to the failure of β-cell compensation in the face of increased insulin demand.

In the present study, *Gcg* ^*gfp/gfp*^ mice fed HFD showed resistance to diet-induced obesity. Energy expenditure in *Gcg* ^*gfp/gfp*^ mice fed HFD was higher than that in *Gcg* ^*gfp/+*^ mice on an HFD, and expression of *Ucp1* and *Dio2* were markedly increased in the BAT of *Gcg* ^*gfp/gfp*^ mice on the HFD. These results suggest that proglucagon-derived peptides promote diet-induced obesity through unknown mechanisms. The exact role of proglucagon-derived peptides in diet-induced obesity is still poorly understood. Previous studies on the effect of exogenous glucagon administration on energy expenditure have suggested that glucagon has beneficial effects on the regulation of body weight and energy expenditure [17, 18]. We have also recently reported that endogenous glucagon is essential for adaptive thermogenesis [21]. Furthermore, HFD-induced body weight gain in *Gcgr* ^*-/-*^ mice was lower than control mice due to the reduction of energy intake [13]. Although several line of evidence suggests that GLP-1 plays some roles in resistance to diet-induced obesity, *Glp1r* ^*-/-*^ mice exhibited resistance to diet-induced obesity due to enhanced physical activity and increased energy expenditure [35, 36]. Studies using administration of GLP-1 agonists and/or antagonists have also provided controversial results on the role of GLP-1 in energy expenditure [37–40]. Our findings, therefore, provide a possibility that combined action of PGDPs is involved in diet-induced obesity via the suppression of BAT activity.

We showed that HFD-feeding induced browning of iWAT in *Gcg* ^*gfp/gfp*^ mice but not in *Gcg* ^*gfp/+*^ mice, and that inflammation of iWAT in *Gcg* ^*gfp/gfp*^ mice was much less severe than in *Gcg* ^*gfp/+*^ mice. Brown-like adipocytes develop in WAT in response to cold exposure or β-adrenergic stimulation [41]. While induction of browning of WAT by HFD, has not been widely recognized, it was recently reported that prolactin deficient mice exhibit brite adipocytes development in perirenal WAT upon HFD-feeding [42]. The mechanisms underlying the induction of iWAT browning under HFD-feeding remain unknown. Recently, Sakamoto et al. reported that inflammation induced by RAW macrophages suppresses *Ucp1* mRNA induction in 10T1/2 adipocytes [43]. Our results therefore suggest that the depletion of proglucagon-derived peptides suppresses the inflammation in iWAT, thereby inducing browning of WAT under HFD-feeding.

In the present study, we showed that mice deficient in proglucagon-derived peptides are resistant to diet-induced obesity but develop diabetes mellitus due to defective β-cell compensation, indicating that glucagon is not a prerequisite for development of diet-induced diabetes. Furthermore, our results demonstrate that GLP-1 is responsible for β-cell compensation triggered by increased insulin demand. Intriguingly, the absence of PGDPs appears to play a pivotal role in facilitating resistance to diet-induced obesity, increased energy expenditure, conserved BAT function, and browning of inguinal WAT. Here, we have unveiled novel aspects of the physiological action of PGDPs, particularly in the functional regulation of adipose tissue.

## Supporting Information

S1 Fig Representative section from *Gcg* ^*gfp/+*^ (upper) and *Gcg* ^*gfp/gfp*^ (lower) mice. Sections were immunostained for insulin (red) and shown with autofluororescence of GFP (green). NCD, mice fed a normal-chow diet; HFD, mice fed a high-fat-diet.
(TIFF)

S2 Fig Pancreatic weight. (A) Pancreatic weight (B) Pancreatic weight is shown as pancreatic weight relative to body weight (BW). White bars, mice fed NCD; black bars, mice fed HFD (n = 5–6). *p < 0.05; ***p < 0.001. Data are presented as means ± SEM.
(TIFF)

S3 Fig β-cell mass (%). β-cell mass (mg) is shown as β-cell area (%) multiplied by pancreatic weight. White bars, mice fed NCD; black bars, mice fed HFD (n = 5–6). ***p < 0.001. Data are presented as means ± SEM.
(TIFF)

S4 Fig Body weight gain on HFD. ΔBW is shown as the difference of body weight (BW) between mice on NCD and mice on HFD at each time point. Open circles, Gcg^gfp/+^ mice; closed circles, Gcg^gfp/gfp^ mice (n = 5–6). *p < 0.05; **p < 0.01; ***p < 0.001. Data are presented as means ± SEM.
(TIFF)

## References

[1] Li XC, Zhuo JL (2013) Current insights and new perspectives on the roles of hyperglucagonemia in non-insulin-dependent type 2 diabetes. Curr Hypertens Rep 15: 522–530 10.1007/s11906-013-0383-y 23996678 PMC3810031

[2] Basu A, Alzaid A, Dinneen S, Caumo A, Cobelli C, Rizza RA (1996) Effects of a change in the pattern of insulin delivery on carbohydrate tolerance in diabetic and nondiabetic humans in the presence of differing degrees of insulin resistance. J Clin Invest 97: 2351–2361 8636416 10.1172/JCI118678 PMC507316

[3] Butler PC, Rizza RA (1991) Contribution to postprandial hyperglycemia and effect on initial splanchnic glucose clearance of hepatic glucose cycling in glucose-intolerant or NIDDM patients. Diabetes 40: 73–81 2015976

[4] Larsson H, Ahren B (2000) Islet dysfunction in insulin resistance involves impaired insulin secretion and increased glucagon secretion in postmenopausal women with impaired glucose tolerance. Diabetes Care 23: 650–657 10834425 10.2337/diacare.23.5.650

[5] Baron AD, Schaeffer L, Shragg P, Kolterman OG (1987) Role of hyperglucagonemia in maintenance of increased rates of hepatic glucose output in type II diabetics. Diabetes 36: 274–283 2879757 10.2337/diab.36.3.274

[6] Shah P, Vella A, Basu A, Basu R, Schwenk WF, Rizza RA (2000) Lack of suppression of glucagon contributes to postprandial hyperglycemia in subjects with type 2 diabetes mellitus. J Clin Endocrinol Metab 85: 4053–4059 11095432 10.1210/jcem.85.11.6993

[7] Jiang G, Zhang BB (2003) Glucagon and regulation of glucose metabolism. Am J Physiol Endocrinol Metab 284: E671–678 12626323 10.1152/ajpendo.00492.2002

[8] Gelling RW, Du XQ, Dichmann DS, Romer J, Huang H, Cui L, et al (2003) Lower blood glucose, hyperglucagonemia, and pancreatic α cell hyperplasia in glucagon receptor knockout mice. Proc Natl Acad Sci U S A 100: 1438–1443 12552113 10.1073/pnas.0237106100 PMC298791

[9] Parker JC, Andrews KM, Allen MR, Stock JL, McNeish JD (2002) Glycemic control in mice with targeted disruption of the glucagon receptor gene. Biochem Biophys Res Commun 290: 839–843 11785978 10.1006/bbrc.2001.6265

[10] Johnson DG, Goebel CU, Hruby VJ, Bregman MD, Trivedi D (1982) Hyperglycemia of diabetic rats decreased by a glucagon receptor antagonist. Science 215: 1115–1116 6278587 10.1126/science.6278587

[11] Van Tine BA, Azizeh BY, Trivedi D, Phelps JR, Houslay MD, Johnson DG, et al (1996) Low level cyclic adenosine 3',5'-monophosphate accumulation analysis of [des-His1, des- Phe6, Glu9] glucagon-NH2 identifies glucagon antagonists from weak partial agonists/antagonists. Endocrinology 137: 3316–3322 8754757 10.1210/endo.137.8.8754757

[12] Winzell MS, Brand CL, Wierup N, Sidelmann UG, Sundler F, Nishimura E, et al (2007) Glucagon receptor antagonism improves islet function in mice with insulin resistance induced by a high-fat diet. Diabetologia 50: 1453–1462 17479245 10.1007/s00125-007-0675-3

[13] Conarello SL, Jiang G, Mu J, Li Z, Woods J, Zycband E, et al (2007) Glucagon receptor knockout mice are resistant to diet-induced obesity and streptozotocin-mediated β cell loss and hyperglycaemia. Diabetologia 50: 142–150 17131145 10.1007/s00125-006-0481-3

[14] Chatzigeorgiou A, Kandaraki E, Papavassiliou AG, Koutsilieris M (2014) Peripheral targets in obesity treatment: a comprehensive update. Obes Rev 10.1111/obr.12163 24612276

[15] Lockie SH, Stefanidis A, Oldfield BJ, Perez-Tilve D (2013) Brown adipose tissue thermogenesis in the resistance to and reversal of obesity: A potential new mechanism contributing to the metabolic benefits of proglucagon-derived peptides. Adipocyte 2: 196–200 10.4161/adip.25417 24052894 PMC3774694

[16] Lo KA, Sun L (2013) Turning WAT into BAT: a review on regulators controlling the browning of white adipocytes. Biosci Rep 33: e00065 10.1042/BSR20130046 23895241 PMC3764508

[17] Habegger KM, Heppner KM, Geary N, Bartness TJ, DiMarchi R, Tschop MH (2010) The metabolic actions of glucagon revisited. Nat Rev Endocrinol 6: 689–697 10.1038/nrendo.2010.187 20957001 PMC3563428

[18] Heppner KM, Habegger KM, Day J, Pfluger PT, Perez-Tilve D, Ward B, et al (2010) Glucagon regulation of energy metabolism. Physiol Behav 100: 545–548 10.1016/j.physbeh.2010.03.019 20381509

[19] Fukami A, Seino Y, Ozaki N, Yamamoto M, Sugiyama C, Sakamoto-Miura E, et al (2013) Ectopic expression of GIP in pancreatic β-cells maintains enhanced insulin secretion in mice with complete absence of proglucagon-derived peptides. Diabetes 62: 510–518 10.2337/db12-0294 23099862 PMC3554360

[20] Hayashi Y, Yamamoto M, Mizoguchi H, Watanabe C, Ito R, Yamamoto S, et al (2009) Mice deficient for glucagon gene-derived peptides display normoglycemia and hyperplasia of islet α-cells but not of intestinal L-cells. Mol Endocrinol 23: 1990–1999 10.1210/me.2009-0296 19819987 PMC5419124

[21] Kinoshita K, Ozaki N, Takagi Y, Murata Y, Oshida Y, Hayashi Y (2014) Glucagon is essential for adaptive thermogenesis in brown adipose tissue. Endocrinology 155: 3484–3492 10.1210/en.2014-1175 24949663

[22] Kanoski SE, Fortin SM, Arnold M, Grill HJ, Hayes MR (2011) Peripheral and central GLP-1 receptor populations mediate the anorectic effects of peripherally administered GLP-1 receptor agonists, liraglutide and exendin-4. Endocrinology 152: 3103–3112 10.1210/en.2011-0174 21693680 PMC3138234

[23] Watanabe C, Seino Y, Miyahira H, Yamamoto M, Fukami A, Ozaki N, et al (2012) Remodeling of hepatic metabolism and hyperaminoacidemia in mice deficient in proglucagon-derived peptides. Diabetes 61: 74–84 10.2337/db11-0739 22187375 PMC3237648

[24] Sakamoto E, Seino Y, Fukami A, Mizutani N, Tsunekawa S, Ishikawa K, et al (2012) Ingestion of a moderate high-sucrose diet results in glucose intolerance with reduced liver glucokinase activity and impaired glucagon-like peptide-1 secretion. J Diabetes Invest 3: 432–440 10.1111/j.2040-1124.2012.00208.x PMC4019243 24843603

[25] Buteau J (2008) GLP-1 receptor signaling: effects on pancreatic β-cell proliferation and survival. Diabetes Metab 34 Suppl 2: S73–77 10.1016/S1262-3636(08)73398-6 18640589

[26] Lee Y, Wang MY, Du XQ, Charron MJ, Unger RH (2011) Glucagon receptor knockout prevents insulin-deficient type 1 diabetes in mice. Diabetes 60: 391–397 10.2337/db10-0426 21270251 PMC3028337

[27] Unger RH, Cherrington AD (2012) Glucagonocentric restructuring of diabetes: a pathophysiologic and therapeutic makeover. J Clin Invest 122: 4–12 10.1172/JCI60016 22214853 PMC3248306

[28] Prentki M, Nolan CJ (2006) Islet β cell failure in type 2 diabetes. J Clin Invest 116: 1802–1812 16823478 10.1172/JCI29103 PMC1483155

[29] Sachdeva MM, Stoffers DA (2009) Minireview: Meeting the demand for insulin: molecular mechanisms of adaptive postnatal β-cell mass expansion. Mol Endocrinol 23: 747–758 10.1210/me.2008-0400 19196831 PMC2691682

[30] Mu J, Jiang G, Brady E, Dallas-Yang Q, Liu F, Woods J, et al (2011) Chronic treatment with a glucagon receptor antagonist lowers glucose and moderately raises circulating glucagon and glucagon-like peptide 1 without severe α cell hypertrophy in diet-induced obese mice. Diabetologia 54: 2381–2391 10.1007/s00125-011-2217-2 21695571

[31] Hansotia T, Maida A, Flock G, Yamada Y, Tsukiyama K, Seino Y, et al (2007) Extrapancreatic incretin receptors modulate glucose homeostasis, body weight, and energy expenditure. J Clin Invest 117: 143–152 17187081 10.1172/JCI25483 PMC1705821

[32] Jun LS, Millican RL, Hawkins ED, Konkol DL, Showalter AD, Christe ME, et al (2015) Absence of glucagon and insulin action reveals a role for the GLP-1 receptor in endogenous glucose production. Diabetes 64: 819–827 10.2337/db14-1052 25288673

[33] Lamont BJ, Li Y, Kwan E, Brown TJ, Gaisano H, Drucker DJ (2012) Pancreatic GLP-1 receptor activation is sufficient for incretin control of glucose metabolism in mice. J Clin Invest 122: 388–402 10.1172/JCI42497 22182839 PMC3248276

[34] Omar BA, Andersen B, Hald J, Raun K, Nishimura E, Ahren B (2014) Fibroblast growth factor 21 (FGF21) and glucagon-like peptide 1 contribute to diabetes resistance in glucagon receptor-deficient mice. Diabetes 63: 101–110 10.2337/db13-0710 24062250

[35] Ayala JE, Bracy DP, James FD, Burmeister MA, Wasserman DH, Drucker DJ (2010) Glucagon-like peptide-1 receptor knockout mice are protected from high-fat diet-induced insulin resistance. Endocrinology 151: 4678–4687 10.1210/en.2010-0289 20685876 PMC2946144

[36] Hansotia T, Baggio LL, Delmeire D, Hinke SA, Yamada Y, Tsukiyama K, et al (2004) Double incretin receptor knockout (DIRKO) mice reveal an essential role for the enteroinsular axis in transducing the glucoregulatory actions of DPP-IV inhibitors. Diabetes 53: 1326–1335 15111503 10.2337/diabetes.53.5.1326

[37] Abegg K, Schiesser M, Lutz TA, Bueter M (2013) Acute peripheral GLP-1 receptor agonism or antagonism does not alter energy expenditure in rats after Roux-en-Y gastric bypass. Physiol Behav 121: 70–78 10.1016/j.physbeh.2013.03.027 23562866 PMC3745783

[38] Bradley DP, Kulstad R, Racine N, Shenker Y, Meredith M, Schoeller DA (2012) Alterations in energy balance following exenatide administration. Appl Physiol Nutr Metab 37: 893–899 10.1139/h2012-068 22735035 PMC3623676

[39] Knauf C, Cani PD, Ait-Belgnaoui A, Benani A, Dray C, Cabou C, et al (2008) Brain glucagon-like peptide 1 signaling controls the onset of high-fat diet-induced insulin resistance and reduces energy expenditure. Endocrinology 149: 4768–4777 10.1210/en.2008-0180 18556349

[40] Osaka T, Endo M, Yamakawa M, Inoue S (2005) Energy expenditure by intravenous administration of glucagon-like peptide-1 mediated by the lower brainstem and sympathoadrenal system. Peptides 26: 1623–1631 16112402 10.1016/j.peptides.2005.02.016

[41] Peschechera A, Eckel J (2013) "Browning" of adipose tissue—regulation and therapeutic perspectives. Arch Physiol Biochem 119: 151–160 10.3109/13813455.2013.796995 23721302

[42] Auffret J, Viengchareun S, Carre N, Denis RG, Magnan C, Marie PY, et al (2012) Beige differentiation of adipose depots in mice lacking prolactin receptor protects against high-fat-diet-induced obesity. FASEB J 26: 3728–3737 10.1096/fj.12-204958 22637534

[43] Sakamoto T, Takahashi N, Sawaragi Y, Naknukool S, Yu R, Goto T, et al (2013) Inflammation induced by RAW macrophages suppresses UCP1 mRNA induction via ERK activation in 10T1/2 adipocytes. Am J Physiol Cell Physiol 304: C729–738 10.1152/ajpcell.00312.2012 23302779 PMC3625802

