# Supplementary figures and images for: Mice Deficient in Proglucagon-Derived Peptides Exhibit Glucose Intolerance on a High-Fat Diet but Are Resistant to Obesity
Source: PLoS One. 2015 Sep 17;10(9):e0138322. doi: 10.1371/journal.pone.0138322 (PMC4574859; doi:10.1371/journal.pone.0138322)

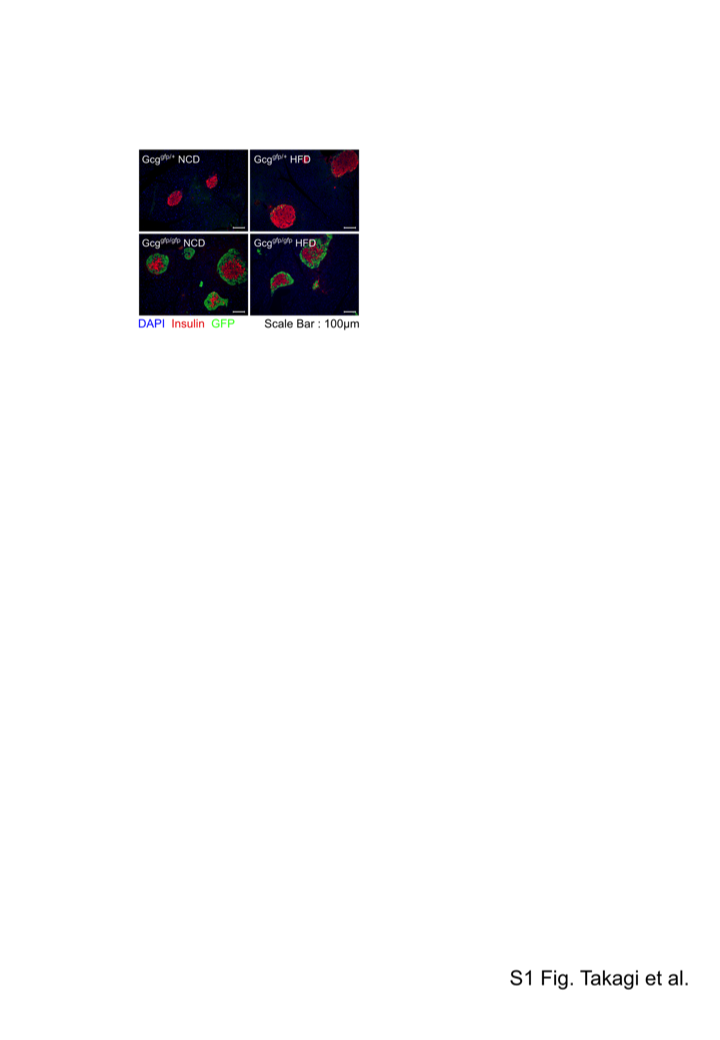

Supplement: S1 Fig — Sections were immunostained for insulin (red) and shown with autofluororescence of GFP (green). NCD, mice fed a normal-chow diet; HFD, mice fed a high-fat-diet. (TIFF) [file pone.0138322.s001.tiff]

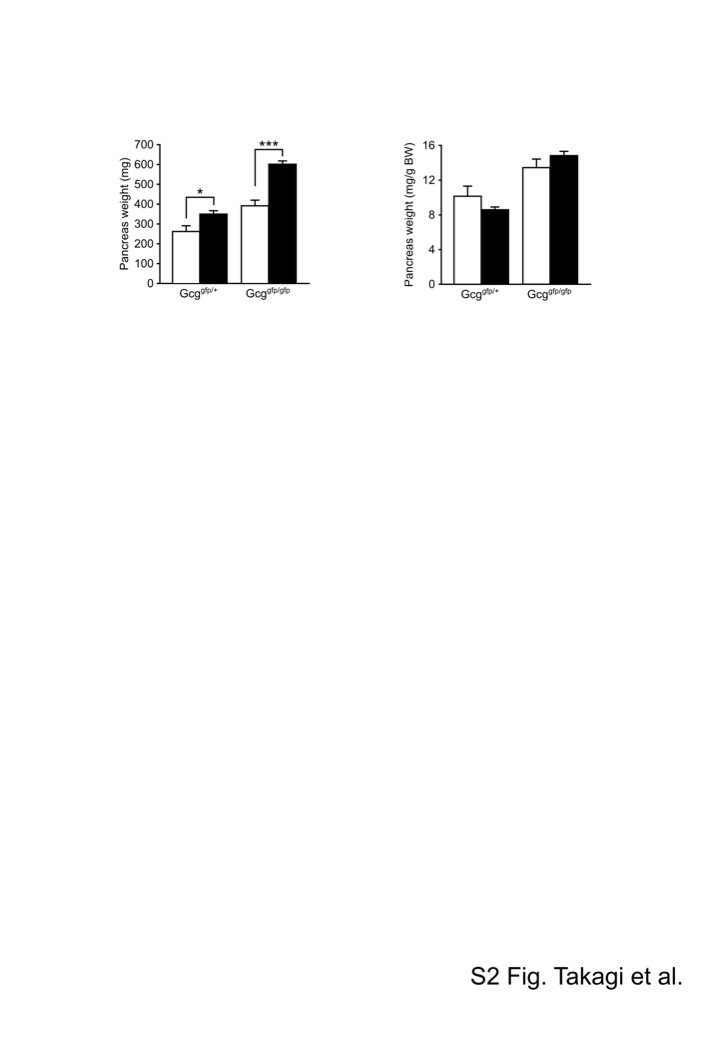

Supplement: S2 Fig — (A) Pancreatic weight (B) Pancreatic weight is shown as pancreatic weight relative to body weight (BW). White bars, mice fed NCD; black bars, mice fed HFD (n = 5–6). *p < 0.05; ***p < 0.001. Data are presented as means ± SEM. (TIFF) [file pone.0138322.s002.tiff]

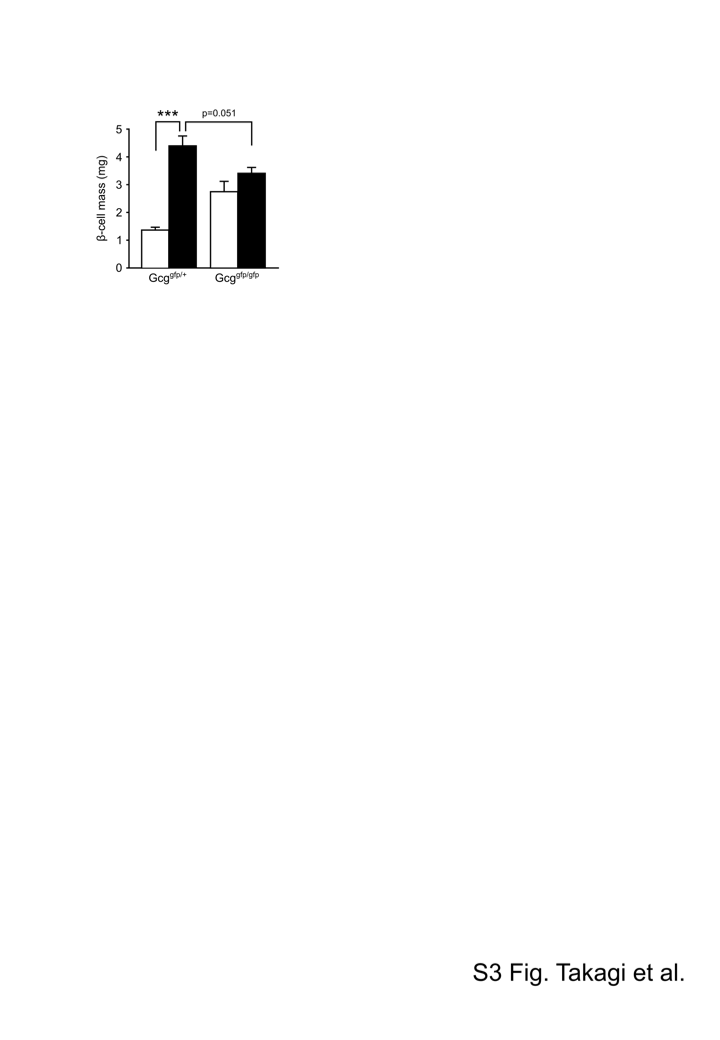

Supplement: S3 Fig — β-cell mass (mg) is shown as β-cell area (%) multiplied by pancreatic weight. White bars, mice fed NCD; black bars, mice fed HFD (n = 5–6). ***p < 0.001. Data are presented as means ± SEM. (TIFF) [file pone.0138322.s003.tiff]

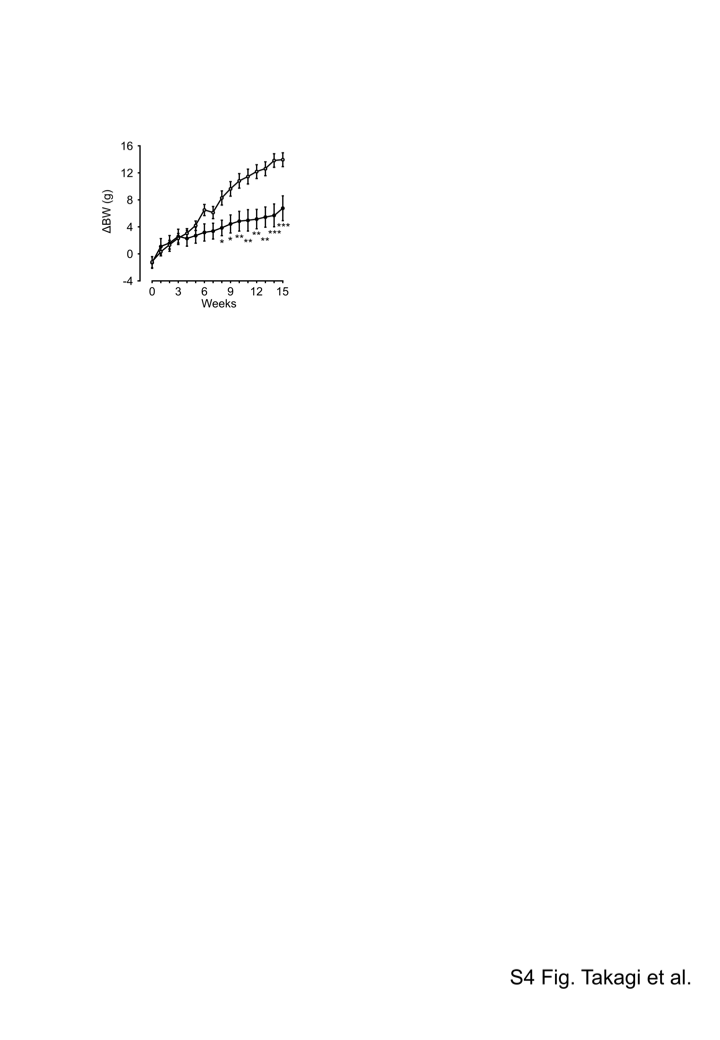

Supplement: S4 Fig — ΔBW is shown as the difference of body weight (BW) between mice on NCD and mice on HFD at each time point. Open circles, Gcggfp/+ mice; closed circles, Gcggfp/gfp mice (n = 5–6). *p < 0.05; **p < 0.01; ***p < 0.001. Data are presented as means ± SEM. (TIFF) [file pone.0138322.s004.tiff]
